# Consequences of the Accidental Introduction of Pieces of Glass into the Body

**Published:** 1843-04

**Authors:** 


*Consequences of the Accidental Introduction of pieces of Glass into the Body.*—Eck, of Berlin, reports in the "Medic. Zeitung," 1842, No. 32, that a Prussian subaltern officer was affected with a partial paralysis of the right arm, which had resisted all the general and local means of treatment employed for its removal. This paralysis, which chifly exhibited itself in the flexor muscles, had been preceded in its commencement by sharp pains, extending from the palmar surface of the thumb along the forearm and humerus. Eck examined the thumb on its palmar side, and on observing several old cicatrices there, he elicited from the patient that a few years previously he had fallen down with a bottle in his hand, several fragments of which had penetrated his thumb; but, as he had been assured, every one of these was afterwards extracted. Eck, however, rationally conceiving that some fragment might still remain to keep up the present symptoms, pressed each of the cicatrices with some force, which operation in one place caused acute pain. He accordingly made a deep incision in that place, and on probing it with the end of a bistoury he found his instrument distinctly to strike against a hard and gritty substance. After the hæmorrhage had been in some degree assuaged, Eck, who now made out clearly that this substance was a piece of glass, extracted it by the help of a pair of forceps, dressed with charpie; but with considerable difficulty, so deeply was it imbedded, and closely enveloped with the surrounding structures. It proved to be about half an inch in length, and of a curvilinear shape, its larger extremity having been the more deeply seated. On its removal the patient soon recovered the complete use of his arm.

In the "Gazette des Hôpitaux" for the 22d Dec. ult., is detailed the case of a man who having severely cut himself by treading on some broken glass in his bedroom, entered the Hotel Dieu, Paris, where his wound was healed, the continuance of a piece of glass, deeply seated within it, not being recognised; but about five months afterwards he was unable from pain to put his foot to the ground, and he re-entered the hospital. Breschet, after ascertaining the fact that a foreign body still remained within the foot, made a crucial incision in the sole, and extracted a piece of glass, nearly an inch in length by half an inch in breadth, from the space between the first and second metatarsal bones. A severe attack of phlebitis supervened after the operation, as far upwards as the groin, and which was not overcome without much care, nor until the lapse of nearly a month, the patient being of a lymphatico-nervous temperament.—*Ibid*, Jan. 21, 1843.

